# Tiprotec preserves endothelial function after cold ischemia and warm reperfusion: comparison between Saline, Custodiol and Tiprotec

**DOI:** 10.1186/1749-8090-10-S1-A74

**Published:** 2015-12-16

**Authors:** Veres Gábor, Hegedűs Péter, Harald Schmidt, Tamás Radovits, Raphael Zöller, Matthias Karck, Gábor Szabó

**Affiliations:** 1 Department of Cardiac Surgery, University of Heidelberg, Heidelberg, 69120, Germany; 2 Heart and Vascular Center, Semmelweis University, Budapest, 1220, Hungary

## Background/Introduction

Background: Coronary artery bypass surgery provides excellent patency rates, however the early/late graft failure reduces the long-term benefit of myocardial revascularization.

## Aims/Objectives

We investigated the effectiveness of generally used Saline, Custodiol solutions and a new solution (Tiprotec) at preserving endothelium after cold ischemia and warm reperfusion injury.

## Method

Aortic transplantations were performed in Lewis rats. Aortic arches stored in Saline, Custodiol and Tiprotec solutions for 2 hours, then were transplanted into abdominal aorta. Two, 24 hours and 1 week after transplantation, the implanted grafts were harvested. Endothelium-dependent and-independent vasorelaxations were investigated in organ bath. DNA strand breaks were assessed by TUNEL-method, mRNA expressions by quantitative real-time PCR and the expression of CD-31 and α-SMA by immunochemistry.

## Results

Severely impaired endothelial function and integrity of implanted aortic grafts were shown after 2h in the Saline, Custodiol group (maximal vasorelaxation to acetylcholine: control:91 ± 2%, Saline:26 ± 5%, Custodiol:24 ± 5%, CD31 positive area control:96 ± 2%, Saline:35 ± 13% Custodiol:54 ± 5%, p < 0.05, respectively), however a preserved endothelial function was observed in the Tiprotec group when compared to the Saline and Custodiol group (maximal vasorelaxation:46 ± 7%, CD31 positive area:54 ± 10%, p < 0.05). After 1 week, endothelial function were partially recovered in all groups, however it was significantly better in the Tiprotec group (maximal vasorelaxation to acetylcholine: Saline:42 ± 3%, Custodiol:48 ± 3%, Tiprotec:56 ± 3%, CD31 positive area: Saline:56 ± 5%, Custodiol:54 ± 4%; Tiprotec:83 ± 6%, p < 0.05, respectively). In addition, mRNA levels of Bax, Bcl-2, eNOS, VEGF-2 and caspase-3 were significantly altered in both groups.

## Discussion/Conclusion

Tiprotec appears to be superior for the preservation of endothelial- and smooth muscle cells of bypass graft after cold storage and warm reperfusion in our murine model.

